# ﻿Three new species of *Quadrastichus* Girault (Hymenoptera, Eulophidae) from China with a key to Chinese species

**DOI:** 10.3897/zookeys.1187.111723

**Published:** 2023-12-20

**Authors:** Wen-Jian Li, Cheng-De Li

**Affiliations:** 1 Jiangsu Provincial Key Laboratory of Coastal Wetland Bioresources and Environmental Protection, School of Wetland, Yancheng Teachers University, Yancheng, 224007, China Yancheng Teachers University Yancheng China; 2 School of Forestry, Northeast Forestry University, Harbin, 150040, China Northeast Forestry University Harbin China

**Keywords:** Chalcidoidea, Hymenoptera, identification key, parasitoids, taxonomy

## Abstract

Six species of *Quadrastichus* Girault (Eulophidae: Tetrastichinae) from China are reviewed, including three new species: *Q.longiseta***sp. nov.**, *Q.flavomaculatus***sp. nov.**, *Q.longiscapus***sp. nov.** and one new country record, *Q.vacuna* (Walker, 1839). New distributional data for *Q.anysis* (Walker, 1839) and *Q.sajoi* (Szelényi, 1941), and a key to the Chinese species of *Quadrastichus* based on females, are included.

## ﻿Introduction

The genus *Quadrastichus* (Eulophidae: Tetrastichinae) was established by Girault (1913) with *Quadrastichusnigrinotatus* Girault as type species fixed by original designation. *Quadrastichus* Girault is a large genus containing 89 valid species worldwide (Noyes 2019), but only seven species are known from China: *Q.anysis* (Walker, 1839), *Q.pteridis* Graham, 1991, *Q.sajoi* (Szelényi, 1941), *Q.liriomyzae* Hansson & LaSalle, 1996, *Q.citrella* Reina & LaSalle, 2004, *Q.erythrinae* Kim, 2004 and *Q.mendeli* Kim & LaSalle, 2008 (Hansson and LaSalle 1996; Zhu and Huang 2001, 2002; Kim et al. 2004; Reina and LaSalle 2004; Zhang et al. 2007; Feng et al. 2016).

*Quadrastichus* species are widely distributed and can be recognized by the following combination of characteristics: submarginal vein (SMV) with a single dorsal seta (occasionally two setae in aberrant specimens of *Q.vacuna*); mid lobe of mesoscutum usually with one adnotaular seta (two or three setae in a few species and five adnotaular setae at most in *Q.erythrinae*); scutellum with submedian grooves; propodeal spiracles close to metanotum; ovipositor sheaths not, or slightly, extending beyond tip of gaster (Graham 1991). When distinguishing different genera, *Quadrastichus* is similar to *Oomyzus* in having only one dorsal seta on the SMV, however, *Quadrastichus* species have all funicular segments longer than broad compared to species of *Oomyzus*. Actually, this genus is most similar to *Aprostocetus*, especially the subgenus Ootetrastichus, in the reduced number of adnotaular setae and slender body, however, *Quadrastichus* species have only one dorsal seta on the SMV and are non-metallic or weakly metallic compared to species of the subgenus Ootetrastichus.

Species of *Quadrastichus* are parasitoids of Cecidomyiidae (Diptera) and various Coleoptera, although other hosts include Cynipidae, Eulophidae (Kim et al. 2008), and Torymidae (Yegorenkova et al. 2007) (Hymenoptera); Agromyzidae and Tephritidae (Diptera); and Gracillariidae (Lepidoptera). Species of *Quadrastichus* are also associated with galls. Larvae of *Q.sajoi* are also predatory within galls of eriophyid mites (Graham 1991; Hansson and LaSalle 1996). *Quadrastichuserythrinae* was reported from galls on *Erythrinavariegata* L. (Kim et al. 2004).

In this paper, we add four more species, including three new species and one new country record to the Chinese fauna. Also, a key to Chinese species is given based on females.

## ﻿Materials and methods

Specimens were collected by sweeping, yellow-pan trapping and were dissected and mounted (dorsal side up) in Canada Balsam following the method described by Noyes (1982) or glued to triangular cards. Photographs were taken with a CCD digital camera attached to an Olympus BX51 compound microscope (slide-mounted specimens) and Leica M205C microscope (card-mounted specimens). Slide-mounted specimen measurements were made using an eye-piece reticle with an Olympus CX21 compound microscope. Card-mounted specimen measurements were taken using an eye-piece reticle with a Motic SMZ168-B dissecting microscope. In the descriptions below, measurements/ratio in brackets after measurement/ratio ranges refer to the holotype. The terminology follows Graham (1987) and Gibson et al. (1997). The following abbreviations are used:

**F1–4** flagellomeres 1–4;

**POL** minimum distance between lateral ocelli;

**OOL** minimum distance between lateral ocellus and eye margin;

**OD** largest diameter of a lateral ocellus;

**MV** marginal vein;

**STV** stigmal vein;

**SMV** submarginal vein.

All specimens studied in this paper are deposited in the insect collections of Northeast Forestry University (**NEFU**) and Yancheng Teachers University (**YCTU**).

## ﻿Results

### ﻿Key to Chinese species of *Quadrastichus* Girault (females)

**Table:** 

1	Mid lobe of mesoscutum with 2–5 adnotaular setae on each side	**2**
–	Mid lobe of mesoscutum with 1 adnotaular seta on each side	**7**
2	Large fovea below eye present; pronotum with 4 coarsely reticulate yellow areas (Fig. 38)	***Q.sajoi* (Szelényi)**
–	Large fovea below eye absent; pronotum uniformly sculptured	**3**
3	Antenna with scape distinctly extending above vertex; clava slender, 6.3–7.0× as long as broad, terminal spine as long as C3 (Fig. 20)	***Q.longiscapus* sp. nov.**
–	Antenna with scape not extending above vertex; clava shorter, at most 4.0× as long as broad, terminal spine distinctly shorter than C3	**4**
4	Body black without yellow markings; propodeum with a distinct median carina	**5**
–	Body mainly yellow or black with yellow markings; propodeum without a distinct median carina	**6**
5	Clava 2.7–3.0× as long as broad (Fig. 28); gaster 2.5–4.0× as long as broad (Fig. 32)	***Q.vacuna* (Walker)**
–	Clava 3.5–4.0× as long as broad; gaster 2.0–3.0× as long as broad	***Q.pteridis* Graham**
6	Mid lobe of mesoscutum with a distinct median line; hypopygium extending 0.3–0.4× the length of gaster (see Kim et al. 2008: fig. 1)	***Q.mendeli* Kim & La Salle**
–	Mid lobe of mesoscutum with a very weak median line or without line; hypopygium extending 0.8–0.9× the length of gaster (see Kim et al. 2004: fig. 28)	***Q.erythrinae* Kim**
7	Forewing with speculum present and extending below MV; MV 6.2× STV (see Reina and La Salle 2004: fig. 10)	***Q.citrella* Reina & La Salle**
–	Forewing with speculum absent or small, not extending below MV; MV at most 4.4× STV	**8**
8	Antenna with clava more than 4.5× as long as broad	**9**
–	Antenna with clava shorter than 4.5× as long as broad	**10**
9	Mid lobe of mesoscutum without median line; propodeum without paraspiracular carinae (Fig. 3); gaster dark brown without a yellow spot at base (Fig. 9)	***Q.longiseta* sp. nov.**
–	Mid lobe of mesoscutum with a distinct median line; propodeum with distinct paraspiracular carinae (Fig. 13); gaster dark brown with a yellow spot at base (Fig. 18)	***Q.flavomaculatus* sp. nov.**
10	Mid lobe of mesoscutum mainly yellow but with a dark area anteromedially	***Q.liriomyzae* Hansson & LaSalle**
–	Mid lobe of mesoscutum completely dark brown to black (Fig. 34)	***Q.anysis* (Walker)**

#### ﻿ Quadrastichus longiseta sp. nov.

Taxon classificationAnimaliaHymenopteraEulophidae

C4628792-2FDD-54FB-ACBB-7F45F7BCBEA6

https://zoobank.org/FCF285E8-A935-451D-8E2E-B9AB984E6E87

Figs 1–6 , 7, 8 , 9–10

##### Type material.

***Holotype***, female [on card], China, Jiangxi Province, Yichun City, Mt. Guan Shan, 25.VIII.2018, Xiang-Xiang Jin, Wang-Ming Li, by sweeping (deposited in YCTU). ***Paratypes***: 3 females, 1 male. [1 female, 1 male on slides], same data as holotype; [1 female on slide, 1 female on card], same locality as holotype, but collected 24.VIII.2018. All paratypes are deposited in NEFU.

##### Diagnosis.

**Female.** Body mainly dark brown with weak metallic reflections. Antenna with scape just reaching, not extending above vertex, 4.3–4.6× as long as broad; pedicel longer than F1; funicle slender and thickening at base of each each funicular segment, F1 shortest, F2 shorter than or as long as F3, clava distinctly longer than F2 and F3 combined, 6.0–7.0× as long as broad, terminal spine as long as C3, flagellum with numerous curved long setae on each segment. **Male.** Body black with bluish metallic reflection. Antenna with plaque 0.5× the length of scape, flagellum with numerous whorls of long setae at base of each segment, especially on funicular segments.

Following Graham (1991), *Q.longiseta* should belong in the *anysis*-group as follows: body black with yellow markings; frons with median area but without median carina; malar sulcus curved. This species is similar to *Q.anysis* (Walker), but can be separated from this species by the following combination of characters: pedicel longer than F1 (vs as long as); F1 shortest, F2 shorter than or as long as F3 (vs F1–F3 subequal in length); clava distinctly longer than F2 and F3 combined, 6.0–7.0× as long as broad (vs clava as long as F2+F3, 3.2–3.9× as long as broad).

##### Description.

**Female. *Body*** (Figs 9, 10) length 0.9–1.1 mm (1.1 mm). Head dark brown, eyes reddish-white, ocelli white. Antenna with scape yellowish-white; pedicel and flagellum yellowish-brown. Mesosoma dark brown, legs with pro- and metacoxae mainly brown, mesocoxae yellowish with base brown, trochanters, femora, tibiae and basal three tarsomeres yellowish, 4^th^ tarsomere yellowish-brown. Wings hyaline, venation brownish. Gaster dark brown, with 1–3 yellow bands dorsally.

**Figures 1–6.**
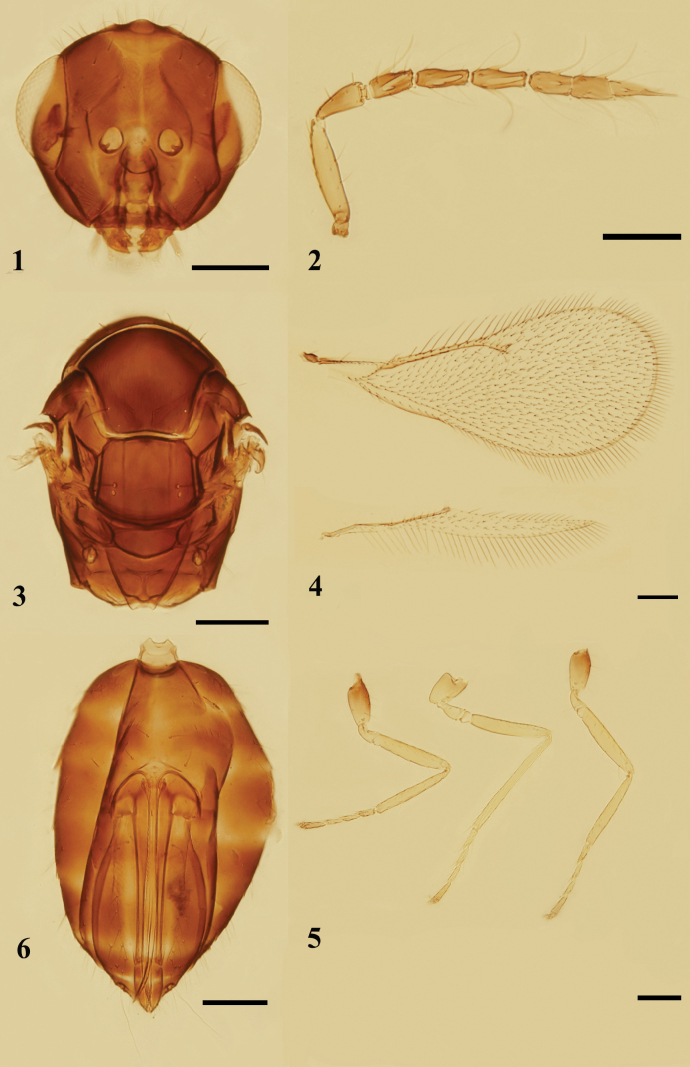
*Quadrastichuslongiseta* sp. nov., paratype, female **1** head, frontal view **2** antenna, lateral view **3** mesosoma, dorsal view **4** fore and hind wings, dorsal view **5** legs, lateral view, from left to right: fore, mid, and hind legs **6** metasoma, ventral view. Scale bars: 100 μm.

***Head*** (Fig. 1) in dorsal view, slightly broader than mesosoma, 2.2× as broad as long. Vertex and face with numerous erect setae, the longest seta as long as OD. Face depressed; frons with a fine median line; POL 1.5–1.6× (1.5×) OOL, OOL 2× OD. Malar sulcus distinctly curved; malar space 0.6× as long as an eye; mouth opening 1.3–1.4× (1.3×) as wide as malar space. Anterior margin of clypeus weakly bidentate, mandibles tridentate. ***Antenna*** (Fig. 2) with lower edge of antennal toruli situated above lever of lower margin of eyes, scape as long as an eye, just reaching but not extending above vertex, 4.3–4.6× (4.5×) as long as broad; pedicel longer than F1, 2.3× as long as broad; 1 discoid anellus; funicle slender and thickening at base, F1 shortest, F2 shorter than or as long as F3, F1–F3: 1.8–2.0× (2.0×), 2.3–2.6× (2.5×) and 2.3–2.8× (2.5×) as long as broad respectively; clava distinctly longer than F2 and F3 combined, as broad as F3, 6.0–7.0× (6.7×) as long as broad, terminal spine as long as C3, flagellomeres with numerous curved long setae, sensilla few.

***Mesosoma*** (Figs 3, 9) 1.4–1.5× (1.4×) as long as broad. Pronotum subconical, arched, reticulation fine. Mid lobe of mesoscutum 1.2–1.3× (1.2×) as broad as long, without median line, with 1 adnotaular seta on each side situated in posterior half, reticulation dense and narrow. Scutellum 1.3× as broad as long; anterior pair of setae attached slightly behind the middle, submedian and sublateral grooves distinct, distance between submedian grooves 1.8–2.0× as broad as distance between submedian grooves and sublateral grooves, reticulation dense. Dorsellum about 2.0× as broad as long. Propodeum medially distinctly longer than dorsellum, reticulation extremely fine; median carina weak, only present in posterior half, without paraspiracular carinae; spiracles round, separated from metanotum by less than their own diameter; callus with 2 or 3 setae. ***Fore wing*** (Fig. 4) 2.2–2.3× (2.3×) as long as broad, costal cell narrow, shorter than MV; MV 3.3–4.0× (3.8×) STV; SMV with 1 dorsal seta; speculum absent; the longest marginal seta slightly longer than STV. ***Hind*** (Fig. 4) wing pointed, 9.4× as long as broad. ***Legs*** (Fig. 5) slender, with meso- and metabasitarsus as long as the corresponding second tarsomere, metafemora 5.1× as long as broad.

**Figures 7, 8.**
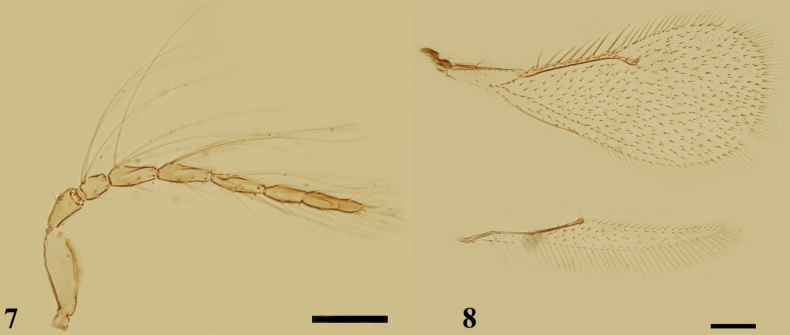
*Quadrastichuslongiseta* sp. nov., paratype, male **7** antenna, lateral view **8** fore and hind wings, dorsal view. Scale bars: 100 μm.

**Figures 9, 10.**
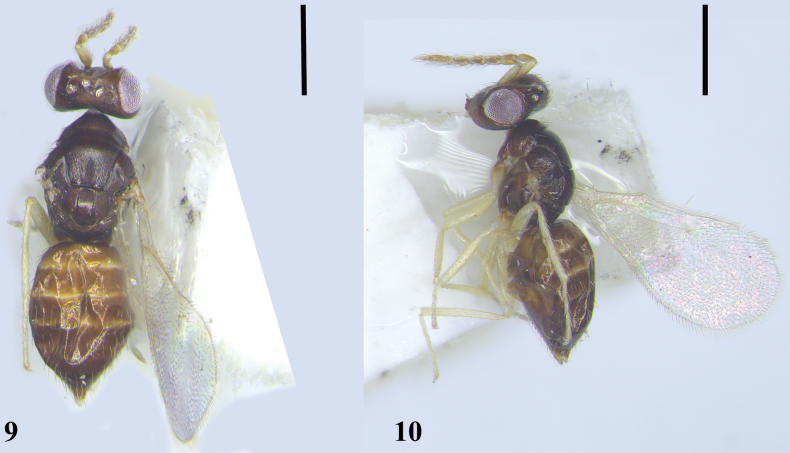
*Quadrastichuslongiseta* sp. nov., holotype, female **9** habitus, dorsal view **10** habitus, lateral view. Scale bars: 300 μm.

***Gastral petiole*** (Fig. 6) present and transverse. ***Gaster ovate*** (Fig. 6), 1.5–1.8× (1.5×) as long as broad, slightly longer and broader than mesosoma, slightly shorter than head and mesosoma combined; each gastral tergite with long erect setae on dorsal surface; each cercus with 3 setae, the longest seta 2.3× as long as the second longest seta; ovipositor 0.7× as long as gaster, ovipositor sheaths extending slightly beyond the tip of gaster; tip of hypopygium situated slightly before middle of gaster.

**Male.** Body length 0.9 mm. Mostly similar to female. ***Antenna*** (Fig. 7) with scape robust, shorter than an eye, 2.5× as long as broad; plaque 0.5× as long as scape; pedicel 1.7× as long as broad; F1–F4: 1.3×, 2.4×, 2.8× and 3.5× as long as broad respectively; each flagellomere with numerous whorls of long setae at base, especially on the funicle. ***Fore wing*** (Fig. 8) with MV 3.5× as long as STV.

##### Host.

Unknown.

##### Distribution.

China (Jiangxi).

##### Etymology.

The epithet *longiseta* refers to the long setae on the antennae in both sexes.

#### ﻿ Quadrastichus flavomaculatus sp. nov.

Taxon classificationAnimaliaHymenopteraEulophidae

0C74D52F-1DCF-5E01-9675-628626842907

https://zoobank.org/BF1B3961-2345-4471-AF35-ECBEADD41C43

Figs 11–16 , 17–18

##### Type material.

***Holotype***, female [on card], China, Shaanxi Province, Ankang City, 5.VIII.2015, Ye Chen, Chao Zhang, by sweeping (deposited in YCTU). ***Paratypes***: 4 females. [1 female on slide and 1 female on card], same data as holotype; [1 female on slide], China, Liaoning Province, Anshan City, 20.IX.2015, Hui Geng, Yan Gao, Xin-Yu Zhang, by sweeping; [1 female on slide], CHINA, Jilin Province, Mt. Changbai Shan, 6.VII.2012, Si-Zhu Liu, Jiang Liu, by sweeping. All paratypes are deposited in NEFU.

##### Diagnosis.

**Female.** Gaster with a yellow spot at base dorsally (Figs 17, 18). Frons with a median line. Antenna slender with pedicel distinctly shorter than each funicle segment. Mid lobe of mesoscutum with median line distinct. Propodeum with median carina and paraspiracular carinae distinct. **Male.** Unknown.

*Quadrastichusflavomaculatus* is similar to *Q.anysis* (Walker), but can be separated from this species by the following combination of characters: pedicel distinctly shorter than F1 (vs as long as); each funicle segment more than 2.5× as long as broad (vs 2.0–2.2×); mid lobe of mesoscutum with a distinct median line (vs weak or absent); gaster 2.7–3.4× as long as broad (vs 1.2–1.8×).

##### Description.

**Female. *Body*** (Figs 17, 18) length 1.4–1.8 mm (1.7 mm). Head dark brown, eyes reddish-white, ocelli white. Antenna with scape mainly yellow, dorsal part yellowish-brown, pedicel and flagellum yellowish-brown. Mesosoma dark brown; legs yellow except brown base of metacoxae. Wings hyaline, venation yellow. Metasoma brown with a yellow spot at base of gaster extending from first to third tergite (Fig. 18).

**Figures 11–16.**
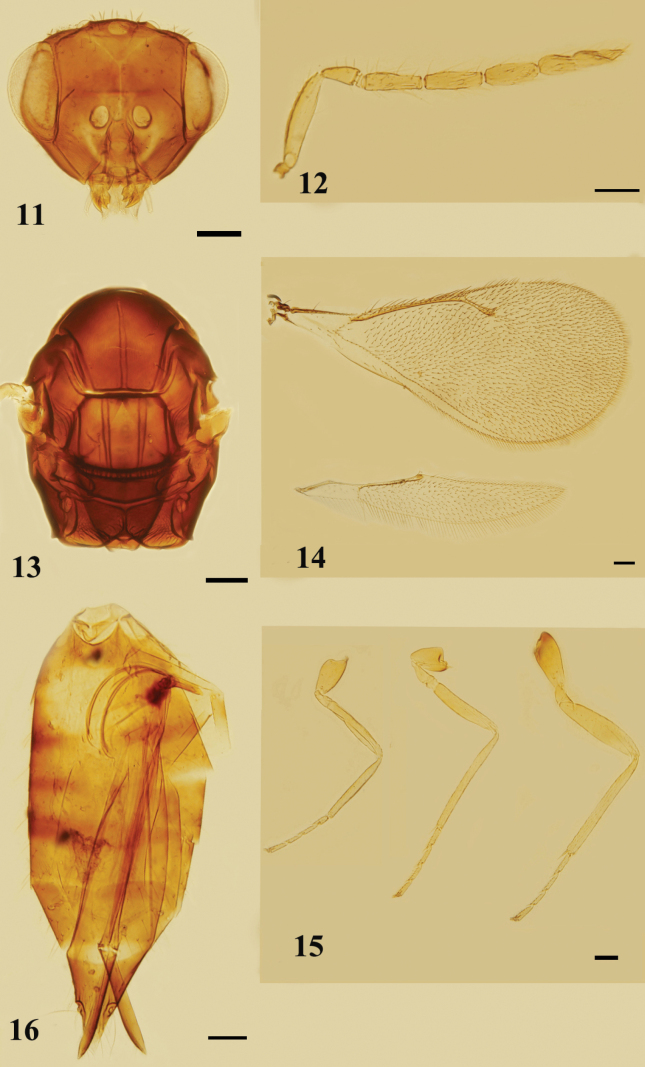
*Quadrastichusflavomaculatus* sp. nov., paratype, female **11** head, frontal view **12** antenna, lateral view **13** mesosoma, dorsal view **14** fore and hind wings, dorsal view **15** legs, lateral view, from left to right: fore, mid, and hind legs **16** metasoma, ventral view. Scale bars: 100 μm.

***Head*** (Fig. 11) in dorsal view as broad as mesosoma, 3.2–3.5× (3.2×) as broad as long. Vertex with short setae, the longest seta slightly shorter than OD. Face depressed; frons with a distinct median line; POL 1.8–2.0× (2.0×) OOL, OOL 1.9–2.0× (1.9×) OD. Malar sulcus slightly curved; malar space 0.55× as long as an eye; mouth opening 1.3× as wide as malar space. Anterior margin of clypeus bidentate, mandibles tridentate. Lower edge of antennal toruli situated above level of ventral edge of eyes. ***Antenna*** (Fig. 12) with scape as long as an eye, reaching but not extending above vertex, 4.0–4.4× (4.2×) as long as broad; 2 anelli; pedicel distinctly shorter than each funicle segment, 2.0–2.2× (2.2×) as long as broad; funicle slender, F1–F3 equal in length, F3 slightly broader than F1 and F2, F1–F3: 3.0–3.2× (3.2×), 2.8–3.2× (3.2×) and 2.6–2.8× (2.6×) as long as broad respectively; clava slightly shorter than or as long as F2 and F3 combined, 4.5–5.5× (5.0×) as long as broad, terminal spine shorter than C3, flagellum with numerous short setae.

***Mesosoma*** (Fig. 13) 1.3–1.5× (1.4×) as long as broad. Pronotum short, arched. Mid lobe of mesoscutum 1.2× as broad as long, median line distinct and complete, with 1 adnotaular seta on each side situated in posterior half, reticulation fine. Scutellum 1.4× as broad as long; anterior pair of setae situated a little behind middle; sublateral grooves distinct, submedian grooves narrow posteriorly, distance between submedian grooves 1.2–1.8× as broad as distance between submedian grooves and sublateral grooves, reticulation fine. Dorsellum about 2.6× as broad as long. Propodeum medially slightly longer than dorsellum, reticulation distinct and dense; median carina and paraspiracular carinae distinct; spiracles round, almost touching hind margin of metanotum; callus with 2 setae. ***Fore wing*** (Fig. 14) 2.1–2.2× (2.1×) as long as broad, costal cell narrow, shorter than MV; MV 3.0–3.2× (3.0×) STV; SMV with 1 dorsal seta; speculum present and small, not extending below MV; marginal setae short. Hind wing (Fig. 14) 5.0× as long as broad. ***Legs*** (Fig. 15) with meso- and metabasitarsus as long as the corresponding second tarsomere, metafemora 4.3–4.6× (4.6×) as long as broad.

***Gastral petiole*** (Fig. 16) transverse. ***Gaster*** (Fig. 16) lanceolate, 2.5–3.5× (2.5×) as long as broad, distinctly longer than mesosoma, 1.2–1.4× (1.4×) the combined length of head and mesosoma; each tergite with long erect setae on dorsal side; each cercus with 3 setae, the longest seta about 2× as long as next longest seta; ovipositor 0.8× as long as length of gaster, ovipositor sheaths extending slightly beyond the tip of gaster; tip of hypopygium situated at anterior 1/3 of gaster.

**Male.** Unknown.

##### Host.

Unknown.

##### Distribution.

China (Jilin, Liaoning, Shaanxi).

##### Etymology.

The epithet *flavomaculatus* refers to the yellow spot at the base of the gaster.

**Figures 17, 18.**
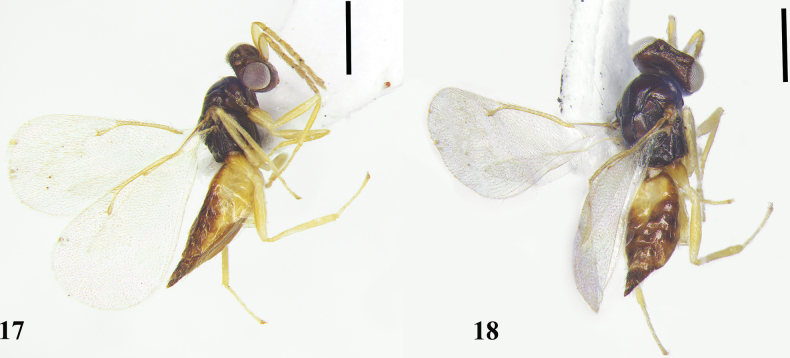
*Quadrastichusflavomaculatus* sp. nov., holotype, female **17** habitus, lateral view **18** habitus, dorsallateral view. Scale bars: 500 μm.

#### ﻿ Quadrastichus longiscapus sp. nov.

Taxon classificationAnimaliaHymenopteraEulophidae

524893D0-6A87-557D-B644-1A6E81F2134A

https://zoobank.org/93AEE74D-65F1-482B-BC20-C11E5DCDF70C

Figs 19–24 , 25–26

##### Type material.

***Holotype***, female [on card], China, Jiangxi Province, Yichun City, Mt. Guan Shan, 21.VIII.2018, Xiang-Xiang Jin, Wang-Ming Li, by sweeping (deposited in YCTU). ***Paratypes***: 7 females. [4 females on cards], same data as holotype, deposited in YCTU. [2 females on slides, 1 female on card], same locality as holotype, but collected 24.VIII.2018, deposited in NEFU.

##### Diagnosis.

**Female.** Mid lobe of mesoscutum and scutellum without reticulation. Antenna with scape distinctly extending above vertex, 4.8–5.0× as long as broad; pedicel shorter than F1, 2.8–3.1× as long as broad; clava distinctly shorter than F2 and F3 combined, as broad as F3, 6.3–7.0× as long as broad, terminal spine as long as C3, flagellomeres with numerous curved long setae.

*Quadrastichuslongiscapus* is similar to *Q.xanthosoma* (Graham), but can be separated from the latter by the following combination of characters: pedicel 2.8–3.1× as long as broad (vs 2.0×); F1 about as long as F2 and F3, 2.8–3.0× (vs F1 longest, 3.0–4.0×); clava 6.3–7.0× as long as broad (vs 3.8–4.0×); body dark brown without yellow markings (vs extensively yellow with blackish markings).

##### Description.

**Female. *Body*** (Figs 25, 26) length 1.7–1.8 mm (1.8 mm). Head with vertex dark brown, face yellow, eyes dark red, ocelli brown. Antenna with scape yellow; pedicel and flagellum brown. Mesosoma wholly dark brown or mainly dark brown with mesoscutum, posterior half of sidelobes of mesoscutum and axilla brownish, legs yellow. Wings hyaline, venation brownish. Metasoma dark brown.

**Figures 19–24.**
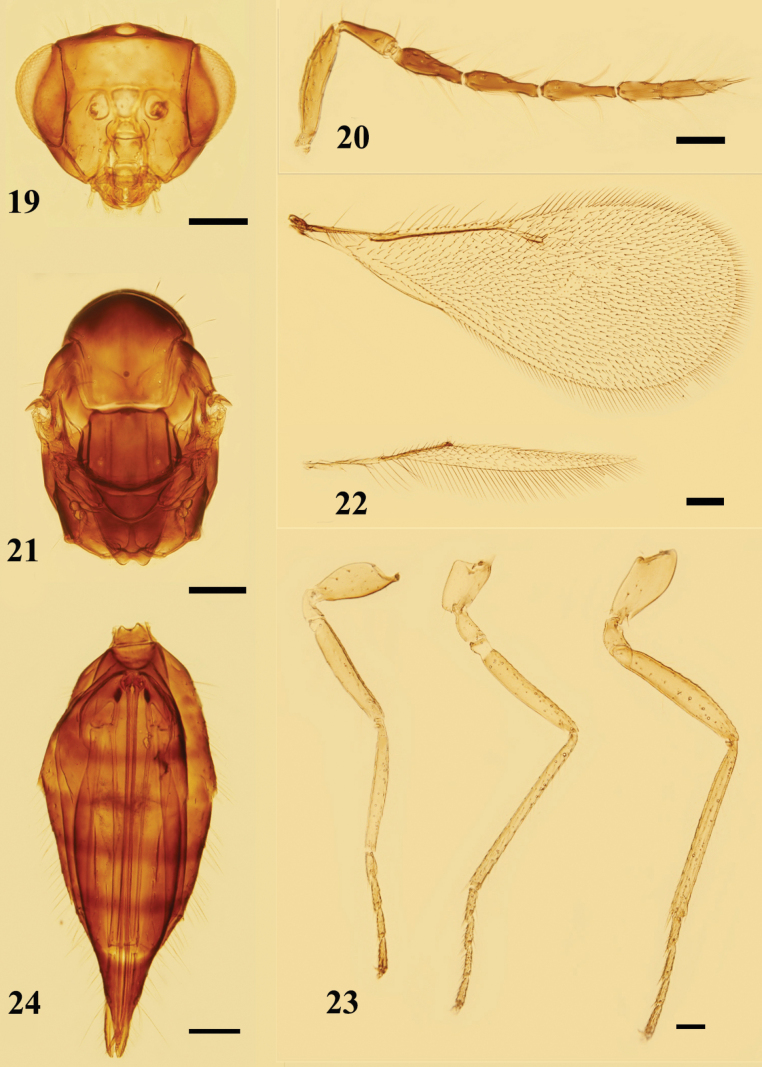
*Quadrastichuslongiscapus* sp. nov., paratype, female **19** head, frontal view **20** antenna, lateral view **21** mesosoma, dorsal view **22** fore and hind wings, dorsal view **23** legs, lateral view, from left to right: fore, mid, and hind legs **24** metasoma, ventral view. Scale bars: 100 μm.

***Head*** (Fig. 19) in dorsal view, slightly broader than mesosoma, 2.8× as broad as long. Vertex and face with numerous erect setae, the longest seta slightly longer than OD. Face depressed; frons without a median line; POL 1.4–1.5× (1.4×) OOL, OOL 2× OD. Malar sulcus distinctly curved; malar space 0.5× as long as an eye; mouth opening 1.7–1.8× (1.8×) as wide as malar space. Anterior margin of clypeus weakly bidentate, mandibles tridentate. ***Antenna*** (Fig. 20) with lower edge of antennal toruli situated above the level of lower margin of eyes, scape as long as an eye, distinctly extending above vertex, 4.8–5.0× (5.0×) as long as broad; pedicel shorter than F1, 2.8–3.1× (3.0×) as long as broad; 1 discoid anellus; funicle slender and thickening at base, F1 about as long as F2 and F3, F1–F3: 2.8–3.0× (3.0×), 3.3–3.8× (3.8×) and 3.2–3.7× (3.7×) as long as broad respectively; clava distinctly shorter than F2 and F3 combined, as broad as F3, 6.3–7.0× (6.8×) as long as broad, terminal spine as long as C3, flagellomeres with numerous curved long setae, sensilla few.

***Mesosoma*** (Fig. 21) 1.4–1.6× (1.4×) as long as broad. Pronotum subconical, arched. Mid lobe of mesoscutum about as broad as long, without median line and reticulation, with 2–3 adnotaular setae on each side. Scutellum 1.3× as broad as long; anterior pair of setae slightly behind middle, submedian and sublateral grooves distinct, distance between submedian grooves 2.0× as broad as distance between submedian grooves and sublateral grooves, without reticulation. Dorsellum long and posterior margin curved down, medially as long as propodeum. Propodeum without reticulation; median carina present and complete, without paraspiracular carinae; spiracles round, separated from metanotum by less than their own diameter; callus with 2 or 3 setae. ***Fore wing*** (Fig. 22) 2.2–2.3× (2.3×) as long as broad, costal cell narrow, shorter than MV; MV 3.1–4.1× (3.4×) STV; SMV with 1 dorsal seta; speculum absent; the longest marginal seta shorter than STV. ***Hind*** (Fig. 22) wing pointed, 12–15× (14.5×) as long as broad. ***Legs*** (Fig. 23) slender, with metabasitarsus slightly shorter than the second tarsomere, metafemora 5.5× as long as broad.

**Figures 25, 26.**
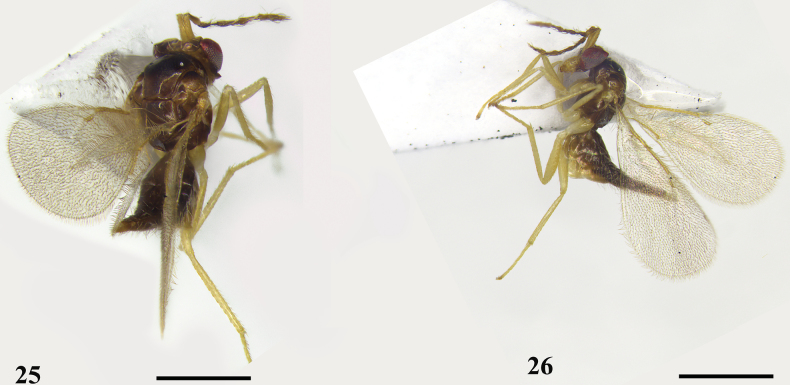
*Quadrastichuslongiscapus* sp. nov., holotype, female **25** habitus, dorsal view **26** habitus, lateral view. Scale bars: 500 μm.

***Gastral petiole*** (Fig. 24) transverse. Gaster lanceolate, 2.1–2.8× (2.3×) as long as broad, distinctly longer than mesosoma, 1.2× as long as head and mesosoma combined; each gastral tergite with numerous long setae on dorsal surface; each cercus with 3 setae, the longest seta 2.0× as long as the second longest seta; ovipositor 0.8× as long as gaster, ovipositor sheaths extending slightly beyond the tip of gaster; tip of hypopygium situated at anterior 1/3 of gaster.

**Male.** Unknown.

##### Host.

Unknown.

##### Distribution.

China (Jiangxi).

##### Etymology.

The epithetic *longiscapus* refers to the long scape of the antennae.

#### ﻿ Quadrastichus vacuna

Taxon classificationAnimaliaHymenopteraEulophidae

(Walker, 1839)

E4F7743E-1C37-5C3D-B377-F599FB5B3744

**Figures 27–32.**
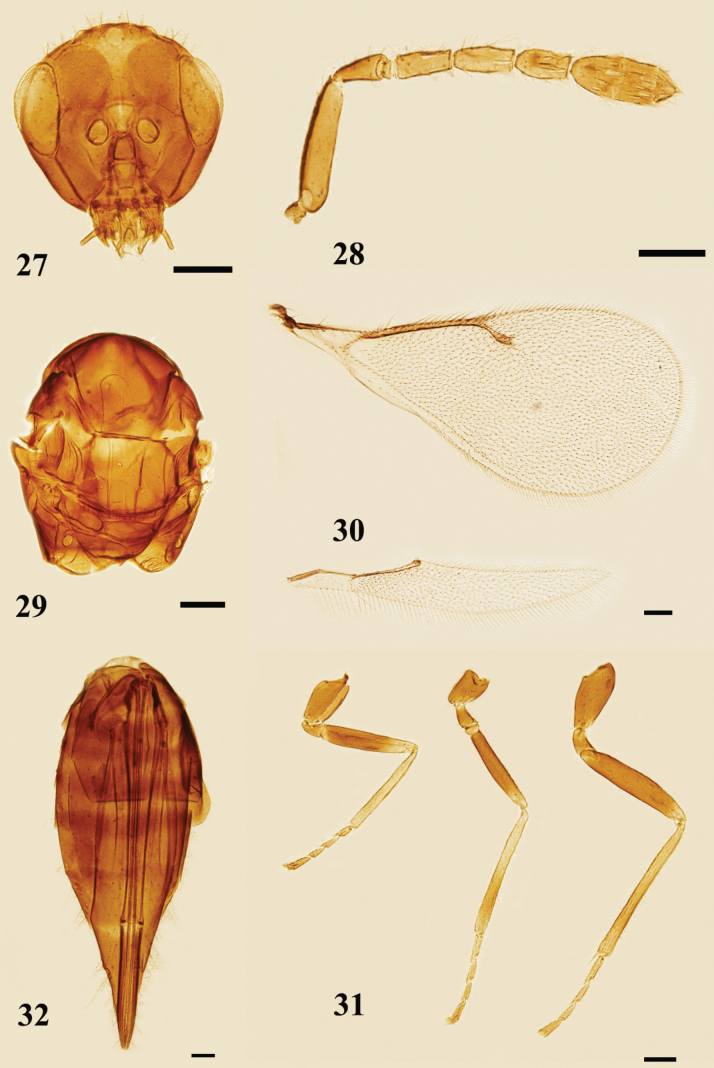
*Quadrastichusvacuna* (Walker), female **27** head, frontal view **28** antenna, lateral view **29** mesosoma, dorsal view **30** fore and hind wings, dorsal view **31** legs, lateral view, from left to right: fore, mid, and hind legs **32** metasoma, ventral view. Scale bars: 100 μm.

**Figure 33.**
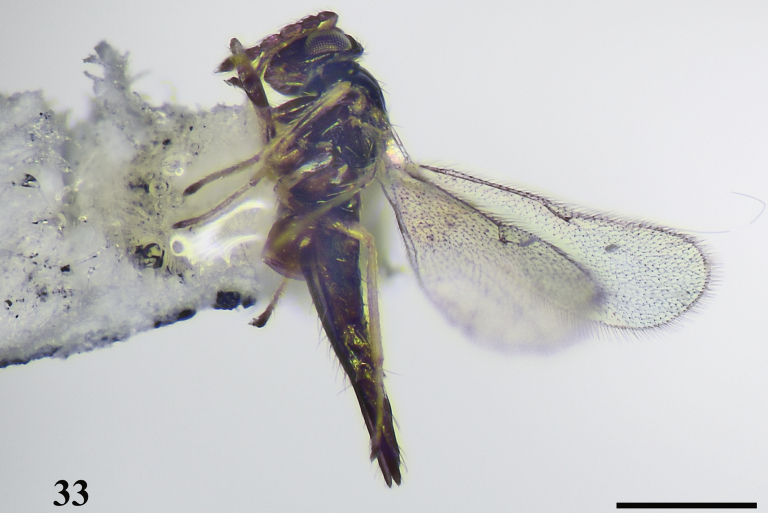
*Quadrastichusvacuna* (Walker), female **33** habitus, lateral view. Scale bar: 500 μm.

Figs 27–32 , 33 New record for China

Cirrospilus vacuna Walker, 1839a: 305.
Cirrospilus numeria Walker, 1839a: 321. [Synonymised by Graham 1961b: 43]
Cirrospilus quercens Walker, 1839a: 307. [Synonymised by Graham 1961b: 43]
Cirrospilusalcithoë Walker, 1839b: 416. [Synonymised by Graham 1961b: 43]
Cirrospilus rhoesus Walker, 1839b: 417. [Synonymised by Graham 1961b: 43]
Cirrospilus sotades Walker, 1839b: 417. [Synonymised by Graham 1961b: 43]
Cirrospilus brunchus Walker, 1840: 236. [Synonymised by Graham 1961b: 43]
Tetrastichus quercens : Walker 1846: 76.
Tetrastichus alcithoe : Walker 1846: 78.
Tetrastichus rhoesus : Walker 1846: 78.
Tetrastichus vacuna : Walker 1848: 149.
Tetrastichus numeria : Walker 1848: 150.
Tetrastichus brunchus : Walker 1848: 151.
Tetrastichus migrator Fӧrster, 1861: 38. [Synonymised by Graham 1961b: 43]
Tetrastichus penetrans Fӧrster, 1861: 38. [Synonymised by Graham 1961b: 43]
Tetrastichus compressiventris Thomson, 1878: 286. [Synonymised by Graham 1961b: 43]
Aprostocetus vacuna : Graham 1961: 42.
Quadrastichus vacuna : Graham and LaSalle 1991: 94.
Qudrastichus vacuna : Boyadzhiev 2003: 82. [Misspelling]

##### Material examined.

5 females: [3 females on cards], China, Shangdong Province, Qindao City, Mt. Lao Shan, 11–13.VII.2014, Guo-Hao Zu, Zhi-Guang Wu, Hai-Feng Bai, by yellow-pan trapping, deposited in YCTU; [1 female on card, 1 female on slide], China, Xizang Province, Linzhi City, Mt. Sejila Shan, 20–24.VIII.2014, Hui- Lin Han, by yellow-pan trapping, deposited in NEFU.

##### Diagnosis.

**Female.** Body (Fig. 33) black without yellow markings. Antenna (Fig. 28) with scape nearly reaching vertex but not extending beyond it, clava 2.7–3.0× as long as broad. Mid lobe of mesoscutum (Fig. 29) with 2–3 adnotaular setae on each side. Propodeum with median carina and paraspiracular carinae. Gaster lanceolate, acuminate, 2.5–4.0× as long as broad, distinctly longer than head and mesosoma combined.

**Male.** Unknown for Chinese material.

##### Hosts.

Unknown from China. Non-Chinese records include *Dasineuraulmariae* (Graham 1991) and *Leucopterascitella* (Herting 1975) (Diptera: Cecidomyiidae) and *Heterarthrusvagans* (Askew and Shaw 1974) (Hymenoptera: Tenthredinidae).

##### Distribution.

China (Xizang, Shandong), Russia, Italy, Hungary, Bulgaria (Boyadzhiev 2003), Netherlands (Gijswijt 2003), Romania (Hansson 2016), Austria, Norway, Poland, Switzerland, Sweden, Czechoslovakia, France, England, Ireland, (Graham 1991).

##### Comments.

Graham (1991) reported that this species occasionally has two dorsal setae on the SMV. However, all specimens we examined only have one dorsal seta on the SMV.

#### ﻿ Quadrastichus anysis

Taxon classificationAnimaliaHymenopteraEulophidae

(Walker, 1839)

34F181FA-45D3-56B7-958D-79A6B1145437

Fig. 34

Cirrospilus anysis Walker, 1839c: 203.
Tetrastichus anysis : Walker 1846: 74.
Aprostocetus anysis : Graham 1961b: 42.
Quadrastichus anysis : Graham and LaSalle 1991: 94; Zhu and Huang 2002: 598.

##### Material examined.

6 females: [2 females on cards], China, Jiangxi Province, Yichun City, Mt. Guan Shan, 21.VIII.2018, Xiang-Xiang Jin, Wang-Ming Li, by sweeping, deposited in YCTU; [1 female on card], China, Heilongjiang Province, Hegang City, Park Beishan, 22.VII.2020, Ming-Rui Li, by sweeping, deposited in YCTU; [2 females on slides], China, Liaoning Province, Anshan City, Mt. Qian Shan, 18.VI.2015, Hui Geng, Yan Gao, by sweeping, deposited in NEFU; [1 female on slide], China, Shaanxi Province, Ankang City, Town Guanghuojie, 3. VIII.2015, Ye Chen, Chao Zhang, by sweeping, deposited in NEFU.

##### Diagnosis.

**Female.** Face with median area but without median carina. Malar sulcus curved. Antenna with scape reaching vertex, about as long as an eye, pedicel 2.0–2.3× as long as broad; F1–F3 equal in length, each 2.0–2.2× as long as broad; clava 3.0–4.0× as long as broad. Mid lobe of mesoscutum with 1 adnotaular seta on each side situated in posterior half, without median line. Propodeum with median carina. Fore wing 2.0–2.1× as long as broad, costal cell narrow, shorter than MV; MV 3.0–4.0× STV; SMV with 1 dorsal seta; speculum small.

**Figure 34.**
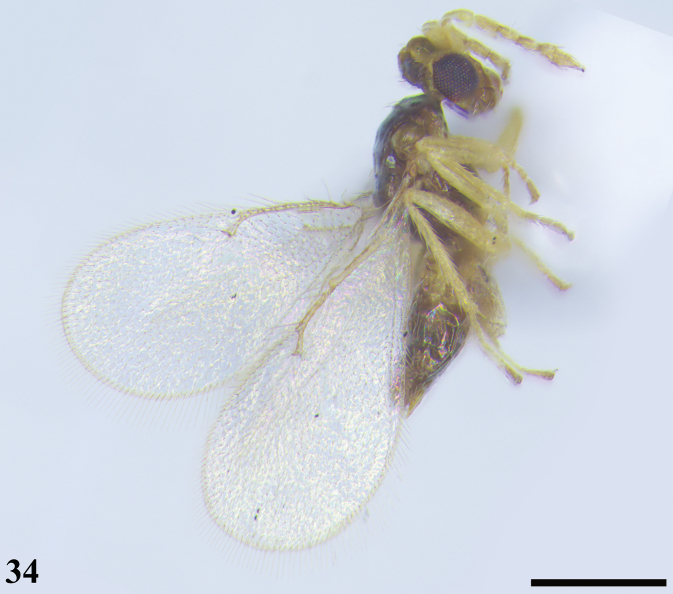
*Quadrastichusanysis* (Walker), female **34** habitus, lateral view. Scale bar: 400 μm.

**Male.** Unknown for Chinese material.

##### Hosts.

Unknown from China. Non-Chinese records include *Monarthropalpusbuxi* (Graham 1991) (Diptera: Cecidomyiidae).

##### Distribution.

China (Beijing, Zhejiang (Zhu and Huang 2001), Gansu, Shaanxi, Guangxi (Zhu and Huang 2002), Heilongjiang, Liaoning, Jiangxi [New records]), Romania, Czechoslovakia, France, Hungary, Italy, England, Russia (Graham 1991).

##### Comments.

Graham (1991) reported that this species was variable in color. The colors of the specimens we examined are also not completely consistent, mainly in the yellow area of gaster, extending from the base to basal half.

#### ﻿ Quadrastichus sajoi

Taxon classificationAnimaliaHymenopteraEulophidae

(Szelényi, 1941)

2D631C2A-7855-5B43-BC2E-A3E39C124E3F

Figs 35 , 36–41

Myiomisasajói Szelényi, 1941: 92. [Justified emendation by Graham 1991: 71]
Tetrastichus sajoi : Vereshchagina 1961: 31.
Aprostocetus scabricollis Graham, 1961a: 18. [Synonymised by Graham 1991: 71]
Quadrastichus sajoi : Graham 1991: 71; Zhu and Huang 2002: 598.
Cecidotetrastichus sajoi : Kostjukov 1997: 800.

##### Material examined.

4 females: [2 females on cards], China, Yunnan Province, Tengchong City, Village Pojiao, 1.V.2013, Xiang-Xiang Jin, Guo-Hao Zu, by sweeping, deposited in YCTU; [1 female on card], China, Jiangxi Province, Yichun City, Mt. Guan Shan, 21.VIII.2018, Xiang-Xiang Jin, Wang-Ming Li, by sweeping, deposited in NEFU; [1 female on slide], China, Jiangxi Province, Yichun City, Mt. Guan Shan, 22.VIII.2018, Xiang-Xiang Jin, Wang-Ming Li, by sweeping, deposited in NEFU.

**Figure 35.**
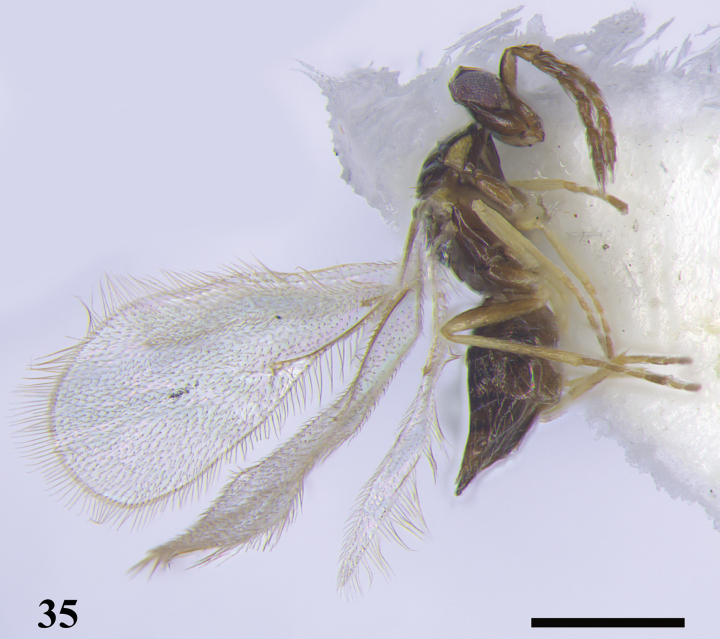
*Quadrastichussajoi* (Szelényi), female **35** habitus, lateral view. Scale bar: 500 μm.

**Figures 36–41.**
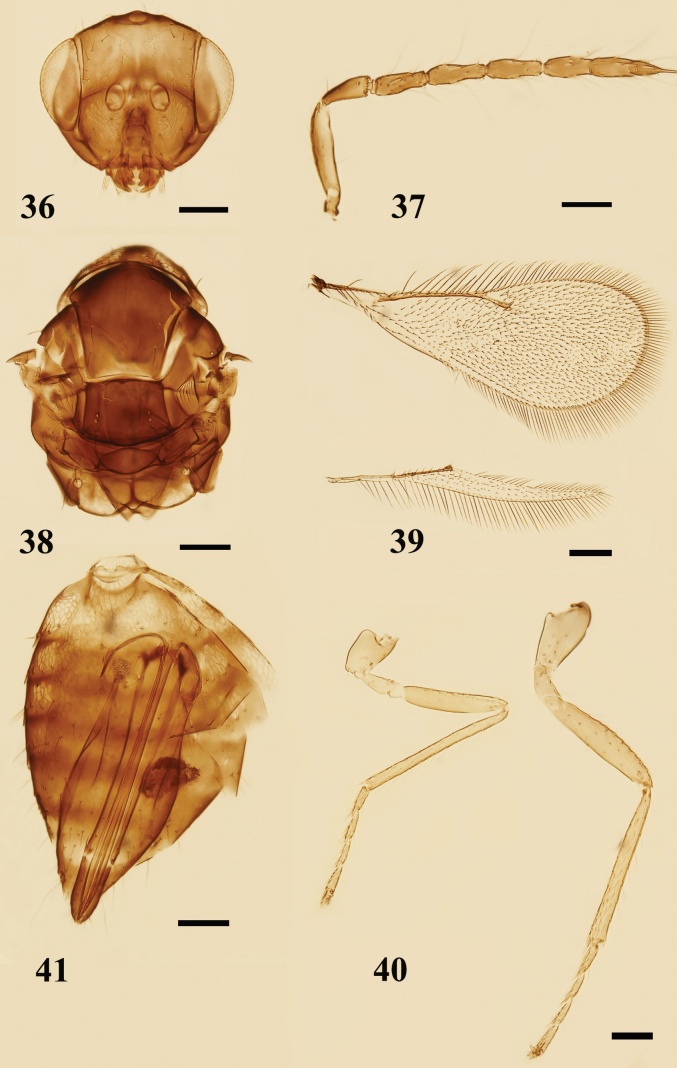
*Quadrastichussajoi* (Szelényi), female **36** head, frontal view **37** antenna, lateral view **38** mesosoma, dorsal view **39** fore and hind wings, dorsal view **40** legs, lateral view, from left to right: mid and hind legs **41** metasoma, ventral view. Scale bars: 100 μm.

##### Diagnosis.

**Female.** Malar sulcus (Fig. 35) distinctly curved, with a large subtriangular fovea below eye. Pronotum (Figs 35, 38) with 4 coarsely reticulate yellowish areas, remaining surface dark. Mid lobe of mesoscutum with 2–3 adnotaular setae on each side. Fore wing (Fig. 39) narrow, 2.2–2.5× as long as broad; MV 3.5–4.0× STV; SMV with 1 dorsal seta; speculum small, marginal setae long.

**Male.** Unknown for Chinese material.

##### Hosts.

Unknown from China. Non-Chinese records include *Acalitusphloeocoptes* (Mezei, 1995) (Acari: Eriophyidae).

##### Distribution.

China (Gansu, Shaanxi, Guangxi (Zhu and Huang 2002), Jiangxi, Yunnan [New records]), Montenegro, Moldova, Serbia, Croatia (Bouček 1977), Syria, Czechoslovakia, Germany, Hungary, Italy, England, Russia (Graham 1991).

##### Comments.

This species can be distinguished from other *Quadrastichus* species by the pronotum with four coarsely reticulate yellowish areas, with the remaining surface dark.

## Supplementary Material

XML Treatment for Quadrastichus longiseta

XML Treatment for Quadrastichus flavomaculatus

XML Treatment for Quadrastichus longiscapus

XML Treatment for Quadrastichus vacuna

XML Treatment for Quadrastichus anysis

XML Treatment for Quadrastichus sajoi

## ﻿Additional information

### Author ORCIDs

Wen-Jian Li https://orcid.org/0009-0004-9790-3608

### Data availability

All of the data that support the findings of this study are available in the main text.
